# Testing the stress gradient hypothesis in soil bacterial communities associated with vegetation belts in the Andean Atacama Desert

**DOI:** 10.1186/s40793-023-00486-w

**Published:** 2023-03-28

**Authors:** Dinka Mandakovic, Constanza Aguado-Norese, Beatriz García-Jiménez, Christian Hodar, Jonathan E. Maldonado, Alexis Gaete, Mauricio Latorre, Mark D. Wilkinson, Rodrigo A. Gutiérrez, Lohengrin A. Cavieres, Joaquín Medina, Verónica Cambiazo, Mauricio Gonzalez

**Affiliations:** 1 Millennium Institute Center for Genome Regulation, Santiago, Chile; 2 Bioinformatic and Gene Expression Laboratory, INTA-Universidad de Chile, Santiago, Chile; 3 GEMA Center for Genomics, Ecology and Environment, Universidad Mayor, Santiago, Chile; 4 Center for Plant Biotechnology and Genomics, Universidad Politécnica de Madrid (UPM)/Instituto Nacional de Investigación y Tecnología Agraria y Alimentaria (INIA)-CSIC, Madrid, Spain; 5 Departamento de Biología, Facultad de Química y Biología, Universidad de Santiago de Chile, 9170022 Santiago, Chile; 6 Laboratorio de Bioingeniería, Instituto de Ciencias de La Ingeniería, Universidad de O’Higgins, Rancagua, Chile; 7 Instituto de Biología Integrativa, Departamento de Genética Molecular y Microbiología, Facultad de Ciencias Biológicas, Pontificia Universidad Católica de Chile, Santiago, Chile; 8 Instituto de Ecología y Biodiversidad (IEB), 4070386 Concepción, Chile; 9 Departamento de Botánica, Facultad de Ciencias Naturales y Oceanográficas, Universidad de Concepción, 4070386 Concepción, Chile; 10 Present Address: Biome Makers Inc., West Sacramento, CA USA

**Keywords:** Plant community, Soil microbiota, Co-occurrence networks, Vegetation belts, Bacteria

## Abstract

**Background:**

Soil microorganisms are in constant interaction with plants, and these interactions shape the composition of soil bacterial communities by modifying their environment. However, little is known about the relationship between microorganisms and native plants present in extreme environments that are not affected by human intervention. Using high-throughput sequencing in combination with random forest and co-occurrence network analyses, we compared soil bacterial communities inhabiting the rhizosphere surrounding soil (RSS) and the corresponding bulk soil (BS) of 21 native plant species organized into three vegetation belts along the altitudinal gradient (2400–4500 m a.s.l.) of the Talabre–Lejía transect (TLT) in the slopes of the Andes in the Atacama Desert. We assessed how each plant community influenced the taxa, potential functions, and ecological interactions of the soil bacterial communities in this extreme natural ecosystem. We tested the ability of the stress gradient hypothesis, which predicts that positive species interactions become increasingly important as stressful conditions increase, to explain the interactions among members of TLT soil microbial communities.

**Results:**

Our comparison of RSS and BS compartments along the TLT provided evidence of plant-specific microbial community composition in the RSS and showed that bacterial communities modify their ecological interactions, in particular, their positive:negative connection ratios in the presence of plant roots at each vegetation belt. We also identified the taxa driving the transition of the BS to the RSS, which appear to be indicators of key host-microbial relationships in the rhizosphere of plants in response to different abiotic conditions. Finally, the potential functions of the bacterial communities also diverge between the BS and the RSS compartments, particularly in the extreme and harshest belts of the TLT.

**Conclusions:**

In this study, we identified taxa of bacterial communities that establish species-specific relationships with native plants and showed that over a gradient of changing abiotic conditions, these relationships may also be plant community specific. These findings also reveal that the interactions among members of the soil microbial communities do not support the stress gradient hypothesis. However, through the RSS compartment, each plant community appears to moderate the abiotic stress gradient and increase the efficiency of the soil microbial community, suggesting that positive interactions may be context dependent.

**Supplementary Information:**

The online version contains supplementary material available at 10.1186/s40793-023-00486-w.

## Background

Soil microorganisms are the most abundant and diverse communities on the planet [1–3] and are the main players in determining ecosystem responses to environmental changes. Soil microbial communities are in constant interaction with plants, which in turn shape the composition of soil microorganisms by influencing their environment [4, 5].

Extensive characterizations of the composition and spatial compartmentalization of soil microbiota have been carried out in crop species [6–10] and model plants [11–13]. However, the characterization of pristine locations from extreme environments provides an opportunity to describe the novel biodiversity of different groups of microbes and determine how they interact with plants. In addition, given the current climate changes and the future predictions of increasing amounts of arid lands [14], it is important to understand how plant-microbial associations change with new environmental conditions [15–17].

Plants have a significant impact on the structure of specific microbial communities associated with their roots [18, 19] since the quantity and composition of root exudates, which are one of the main drivers shaping rhizosphere microbial communities [5, 20], are plant genotype-specific [21, 22]. Plant root exudates provide nutrients for microbial growth and facilitate direct communication between plants and microbes by signaling molecules and phytohormones [23]. Nevertheless, this process is often context dependent, suggesting that many factors may be involved [24], especially under extreme living conditions. For instance, in arid and semiarid regions, plants and soil bacteria have adapted to survive abiotic stresses such as drought, high temperature, and high salinity. A recent study revealed that biological processes enriched among the top-expressed genes of dominant Atacama plant species were related to the stress response (including response to ROS, salt tolerance, DNA reparation, and heat and cold acclimation) and energy production (photosynthesis and cytokine response), among others [25]. In the case of *Hoffmannseggia doellii*, a perennial herb endemic to the Atacama Desert, the recruitment of bacteria with plant beneficial traits to the rhizosphere surrounding soil, including nitrogen fixation, has been reported [26], suggesting that *H.* *doellii*-associated microbiota might be essential for plant growth in zones of low nutrient availability and extreme environmental conditions. On the other hand, quinoa plants, which are known to be well adapted to stressful conditions [27], also depend on their associated microbiota for protection and growth promotion [28, 29].

**Fig. 1:**
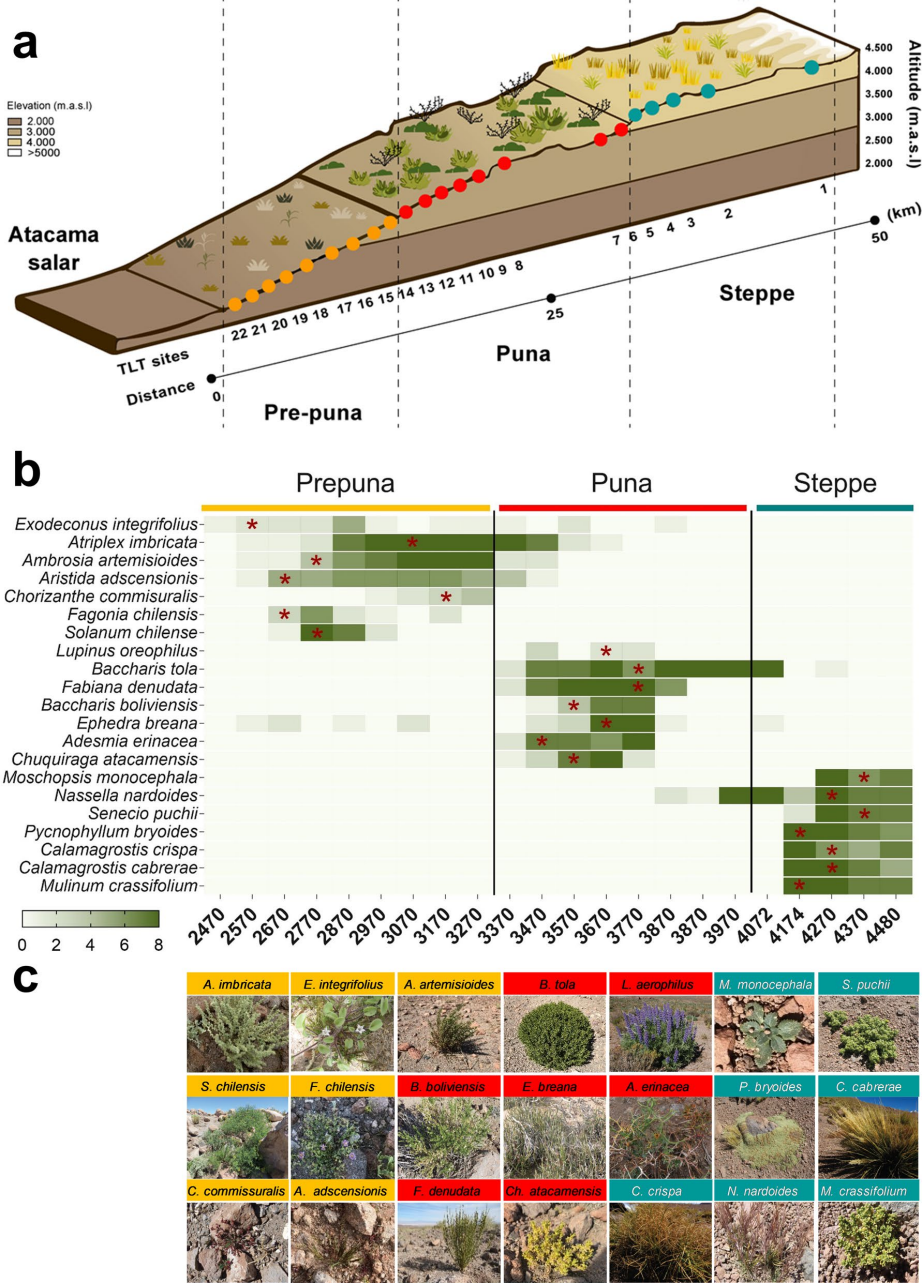
Sampling site and characterization of the plant species across the Talabre–Lejía transect. **a** Elevation model indicating the sampling sites at the TLT. Sampling sites at prepuna (orange dots), puna (red dots) and steppe (green dots) with their corresponding mean annual precipitation (mm) and altitude (m a.s.l.). **b** Distribution of the 21 selected plant species along the TLT elevations. Color gradient represent the number of years each species was observed at each location. Full data on the plants, such as family, growth habit and life history, can be found in Additional file 2: Figure S2 and Additional file 7: Table S1. **c.** Representative images of plant species in each vegetation belt are shown

The Atacama Desert is one of most arid places on Earth [30]. Vegetation is mostly restricted to the western slopes of the Andes range and to the coastal range along the Pacific coast [31, 32]. Along the Andes, there is an altitudinal zonation of vegetation due to decreasing temperature and increasing precipitation with increasing elevation [31, 33]. In contrast to what is expected due to its location within the Atacama Desert, rich plant diversity has been described along an elevational gradient from Laguna Lejia (4500 m a.s.l.) to the eastern margin of the Salar de Atacama (2400 m a.s.l.), hereafter Talabre–Lejía transect (TLT), [25, 34, 35] located at 23.5°S (Additional file 1: Figure S1). Previous studies on the TLT have reported the presence of 78 native plant species [25] distributed in three vegetation belts: (1) “Steppe”, between 4500 and 4000 m above sea level (m a.s.l.), characterized by perennial bunch grasses, cushion plants and subshrubs; (2) “Puna”, between 4000 and 3300 m a.s.l., dominated mostly by shrubs, subshrubs, perennial herbs and several C4 annual grasses, and where the greatest species richness is observed; and (3) “Prepuna”, the lowermost vegetation belt from 3300 to 2400 m a.s.l., where only cushion cacti and subshrubs can withstand the extremely dry conditions of this zone [25, 34, 36] (Fig. 1a). The diversity of plant species along the TLT suggests that their root zone-associated soil microorganisms can be diverse and differentiated within these vegetation belts [25, 34] that could depend, in part, on how bacterial populations interact with one another.

In ecology, the stress gradient hypothesis [37] states that the role of facilitative interactions becomes increasingly important in conjunction with increasing stress. This is because harsh environments may restrict plants from acquiring resources, and any amelioration of these conditions due to the presence of a neighboring plant will favor growth to the extent that it outweighs the negative, competitive impact of growing in close association. Although originally proposed for plants, the stress gradient hypothesis has been tested and extended to animals [38–40] and, more recently, to bacteria [41, 42]. Considering that vegetation is restricted by extremely low temperatures, strong wind, and snowfall at the upper limit of the TLT, whereas at the lower limit of this transect the onset of the absolute desert is marked by almost no precipitation [30, 34], we have a unique and complex gradient of environmental severity where different patterns of ecological interactions can be tested. First, we described and compared soil bacterial communities between the rhizosphere surrounding soil (RSS) and the corresponding bulk soil (BS) of seven native plant species per vegetation belt (defined as plant community), which were selected based on their coverage and frequency along the TLT over a 10-year period [25, 34, 43]. We examined the nature of the ecological interactions that take place in the microbial communities from the BS and the RSS compartments at each vegetation belt and quantified changes in positive (co-occurrence) and negative (exclusion) relationships between members of soil bacterial communities along this stress gradient. Additionally, we used NetShift analysis [44] to identify potential driver taxa based on differences in network co-occurrences between the BS and the RSS of the plant microbial communities. According to the stress gradient hypothesis, we expected an increase in co-occurrences among members of the soil bacterial communities in both the lower and upper vegetation belts.

## Methods

### Sample collection

Representative plant species from the prepuna (n = 7), puna (n = 7) and steppe (n = 7) vegetation belts (Additional file 7: Table S1) were selected based on their coverage (dominance) and frequency [34, 43]. Soil sampling was carried out along TLT (located at 23.5°S, Additional file 1: Figure S1a) from April to May of 2016 and 2017, after the rainy season, which typically takes place from December to March. At each vegetation belt, two different soil compartments were sampled (Additional file 1: Figure S1b): RSS (bacteria loosely attached to the roots) and BS. The RSS compartment was sampled in three different plants per species (biological triplicates, Additional file 1: Figure S1c). Plants were of similar size and developmental stage to avoid physiological differences among sampled plants. Thus, at each vegetation belt, seven plant species were sampled to obtain 21 soil samples. For each plant species, the root system was gently shaken to obtain the RSS fraction (~ 100 g, [26]). BS samples (100 g) were collected in triplicate at 10 cm depth from the ground and at least 1 m away from each sampled plant to obtain 21 soil samples per vegetation belt. All soil samples (n = 126 samples) were immediately stored in dry ice until their arrival at the laboratory, where they were frozen at − 80 °C.

### Soil physicochemical characteristics and nutrient properties

Soil pH measurements were carried out as described by Mandakovic et al. [45]. A portion of the BS and RSS samples was used to determine metal composition using the total reflection X-ray fluorescence (TXRF) technique. Briefly, for each sample, 1 g of soil was resuspended in 1 mL of distilled water and homogenized for 2 h at room temperature. After mixing, the samples were centrifuged at 11,440*g* for 10 min in a Hettich Universal 32R. The soluble fraction was recovered and measured in a Bruker S2 PICOFOX. All these analytical protocols have been previously reported [35, 45]. Pearson’s correlation coefficient was calculated among all nutrients and chemical components that were present in at least 15% of the samples. For those selected variables, missing values were imputed with the missForest R package [46].

### DNA extraction and 16S rRNA amplicon sequencing of BS and RSS from the prepuna, puna and steppe belts

Between 332 and 518 ng/µL of DNA were extracted from 5 g BS and RSS samples and used for posterior sequencing as described by Fernández-Gomez et al. [35]. DNA quantification was performed by fluorometry using the dsDNA Broad-Range Assay Kit (Invitrogen), and sample purity (260/280 ratio) was determined by measuring absorbance using a NanoQuant Infinite M200pro (TECAN). Samples were sequenced by Molecular Research LP (MR DNA) on an Illumina MiSeq platform. Briefly, the microbial 16S rRNA gene was amplified using the bacteria-specific primer set 28F (5′ GA GTT TGA TCM TGG CTC AG 3′) and 519R (5′ GWA TTA CCG CGG CKG CTG 3′) with 2 ng of DNA per reaction [47], with a barcode in the forward primer, and sequenced in an overlapping 2 × 300 bp configuration to obtain a minimum throughput of 40,000 sequences (reads) per sample. All sequence data used in this study have been deposited in the Sequence Read Archive (SRA) of the National Center for Biotechnology Information (NCBI) under BioProject accession number PRJNA489888.

### Processing of Illumina sequence data

The 16S rRNA amplicons were processed and analyzed according to the procedures of Dowd et al. [48] and Handl et al. [49]. Briefly, reads were overlapped by pairs, and barcodes were removed. Sequences < 150 bp or with ambiguous assignations were discarded. Valid sequences were grouped using USearch (v6.1.544) with 4% divergence to remove chimeras and singletons [50, 51]. Finally, sequences were filtered with a minimum quality of 30 (q30) with Mothur v1.22.2 [52].

### Sequence analysis and OTU taxonomic and functional composition

Analysis of the raw 16S rRNA gene amplicon sequence data yielded 131,000,000 total reads after quality trimming. Taxonomic assignation was performed using the software QIIME v1.9.1 [53]. OTUs were identified at 97% identity against the GreenGenes r16S database [54] with USearch v6.1.544 using default parameters in QIIME (sample rarefaction to 8000 reads). The OTUs identified as mitochondrial or chloroplast sequences were removed [35]. We analyzed the microbiota composition using previously reported measures [35]. The mean diversity of species in the different compartments (alpha diversity) was examined using the Chao1 index to estimate the OTU richness based on the relative abundances within samples. Since Chao1 only accounts for richness, we calculated the Shannon index, which summarizes diversity by incorporating the evenness of the samples. All calculations were performed using Mothur software v1.22.2. The Mann–Whitney U test was used to compare the diversity indices, where a *p* value < 0.05 was interpreted to be statistically significant. These tests were calculated in XLSTATS v.2020.3.1.20. Sungear software (http://virtualplant-prod.bio.nyu.edu/cgi-bin/sungear/index.cgi) was used to compare OTU data [55]. Functional prediction was conducted based on 16 S rRNA sequences and the Kyoto Encyclopedia of Genes and Genome (KEGG) database using the PICRUSt2 software package [56]. The differences in the composition of the predicted functions among the RSS and BS samples were detected by linear discriminant analysis effect size (LEfSe).

### Random forest analysis of soil compartments

Random forest [57], a classification machine learning algorithm, was used to identify taxa (OTUs) and nutrients that are relevant to distinguish samples between the two soil compartments (BS and RSS) and among the three different vegetation belts. Then, we computed mean decrease in accuracy (MDA) analysis [46], a random forest-associated metric, where a higher MDA means a higher influence of the feature (taxa or nutrient) to differentiate between groups, soil compartments or vegetation belts. As a default, MDA values are scaled in terms of standard deviation. MDA measures how much of the classification model accuracy is lost when the feature values are permuted. The greater the accuracy suffers when the abundances of taxa change, the more important the taxon is. Thus, a high MDA means that a change in taxa abundance is meaningful to differentiate between soil compartments or vegetation belts.

### Interaction networks based on co-occurrence and exclusions

To examine changes in the composition of the microbial community between the BS and RSS compartments, we generated microbial interaction networks as described by Mandakovic et al. [60] using OTUs as nodes in the networks. Significant co-occurrences or exclusions across the samples were identified by the CoNet method version 1.1.1. beta [58] using a multiple ensemble correlation. Four similarity measures were calculated: Bray‒Curtis and Kullback‒Leibler nonparametric dissimilarity indices and Pearson and Spearman rank correlation coefficients. A distribution of all pairwise scores between OTUs was computed for each site to enrich the network with OTU nodes. Based on OTU distributions, initial thresholds were selected. For each measure and each edge, 100 renormalized permutations and bootstrap scores were generated according to Faust and Raes [58]. Networks were calculated and displayed by Cytoscape version 3.8.2 [59] to reveal the statistics of the networks (number of nodes, number of total interactions, number of positive interactions, number of negative interactions, clustering coefficient, centralization, path length, average neighbors and density).

### Identification of taxa drivers within the RSS and BS co-occurrence networks

We used the NetShift method (https://web.rniapps.net/netshift) to identify potential keystone driver taxa based on differences in network interactions between two predefined states: BS (control) and RSS (case) from each vegetation belt. The driver taxa were identified based on the NESH score (≥ 1.8), which ranks core taxa from both networks according to their connectivity modifications in RSS vs. BS, and the betweenness value (> 0), which indicates the delta betweenness (DelBet) of the core taxa in RSS vs. BS [44].

## Results

### Distribution of plant communities along the TLT

As shown in previous studies [25, 34], the altitudinal gradient of the TLT exhibits distinct vegetation belts, which can be consistently recognized based on overall plant physiognomy (Fig. 1a). We selected seven plant species per belt that had the highest coverage (dominance) and persistence (number of years that the same plant species was detected at each belt after eight years of field trips, Fig. 1b and Additional file 7: Table S1). Most of the selected plants showed a discrete distribution along the TLT, limiting their presence to the limits of their respective vegetation belts (Fig. 1b). Thus, these sets of plant species were considered representative members of the plant communities at each specific vegetation belt along the TLT (Fig. 1c). For soil sampling of each plant species, we defined two compartments according to their proximity to the root system: the RSS (soil and bacteria loosely attached to the root organs) and their corresponding BS (plant-free soil) (Additional file 1: Figure S1b).

### Soil composition of the BS and the RSS compartments along the TLT

The environmental and chemical compositions of the BS and RSS soil compartments were determined in the three TLT vegetation belts (Additional file 7: Table S2) and previously described [25, 45]. Overall, the paired correlations, calculated between the concentrations of nutrients and minerals, showed differences when comparing BS and RSS. In the prepuna belt, the ratios of positive vs. negative correlations observed in both compartments were similar, although differences in the direction of the correlation were detected among certain nutrients or minerals (i.e., Ca, P, K and As). There was a more negative correlation between pairs of nutrients in the BS compartment of the puna than in the RSS compartment. On the other hand, in the steppe belt, we observed major changes in the pattern of correlations between soil nutrients or minerals, with an increase in positive correlations in the BS and in negative correlations in the RSS (Additional file 3: Figure S3). When comparing the BS or the RSS of the three vegetation belts, we observed more negative correlations among nutrients and minerals in the BS compartment of the puna (for example, Fe and Mn) than in the BS of the prepuna and steppe, while in the RSS, the pattern was different, with more negative correlations (P, K, Cu and Ca) in the steppe than in the lower zones of the altitudinal gradient (Additional file 3: Figure S3). Thus, the differences in positive and negative correlation patterns between the BS and the RSS within each vegetation belt suggest that plant roots could influence the nutrient concentration in the soil, whereas differences among the three vegetation belts could be explained by the differential composition of the plant communities across the altitudinal gradient.

### Composition and diversity of the soil bacterial communities along the TLT vegetation belts

**Fig. 2:**
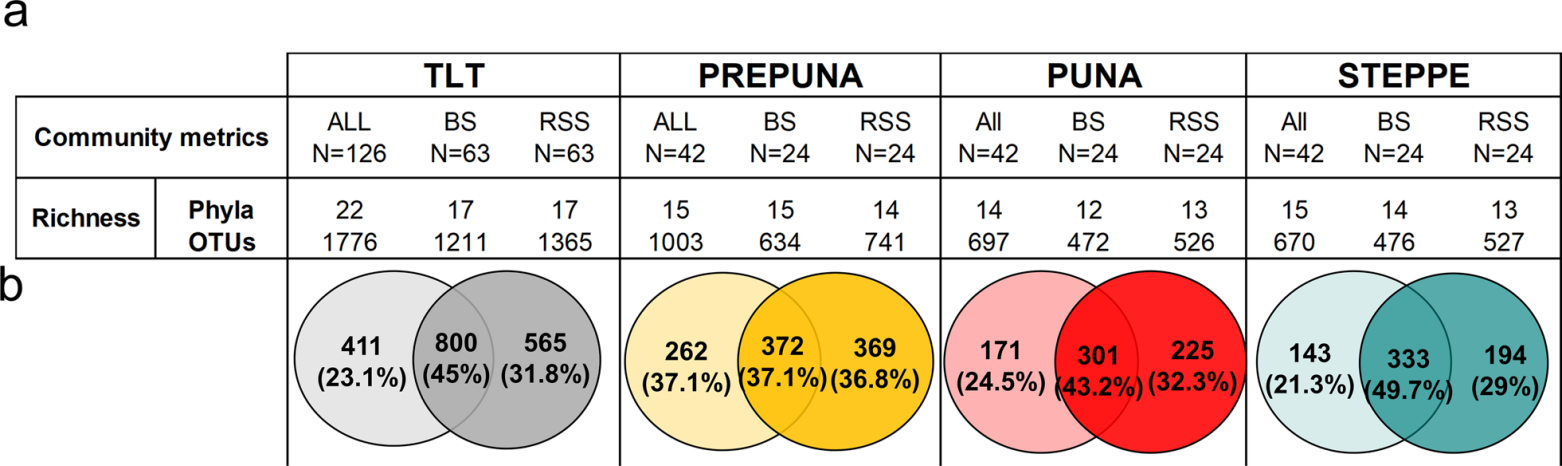
Community metrics of the soil microbiota. **a** Community metrics including phyla, genera, and richness of OTUs of the entire TLT and the number of BS and RSS samples. **b** A Venn diagram showing shared and exclusive OTUs in the BS or RSS compartments (light and dark colors, respectively) in the entire TLT and each vegetation belt (from left to right: TLT; prepuna, puna and steppe)

A total of 14,583 OTUs were obtained from the 126 soil samples. For the following analyses, a subset of 1776 OTUs (Additional file 8: Table S3) were selected based on their relative abundance (more than 0.01%) and consistency (they were identified in at least two of the three replicates of the BS or RSS samples in at least one plant species). This set of OTUs was assigned to 22 phyla (Fig. 2a). Across the vegetation belts, Proteobacteria, Acidobacteria, and Actinobacteria were the most abundant phyla within the BS and RSS communities (Additional file 2: Figure S2). Regarding the diversity data, we did not observe differences in richness, the Shannon index, or phylogenetic diversity of soil microbiota between the BS and the RSS compartments (Additional file 4: Figure S4a) or among the vegetation belts of this altitudinal gradient (Additional file 4: Figure S4b).

**Fig. 3:**
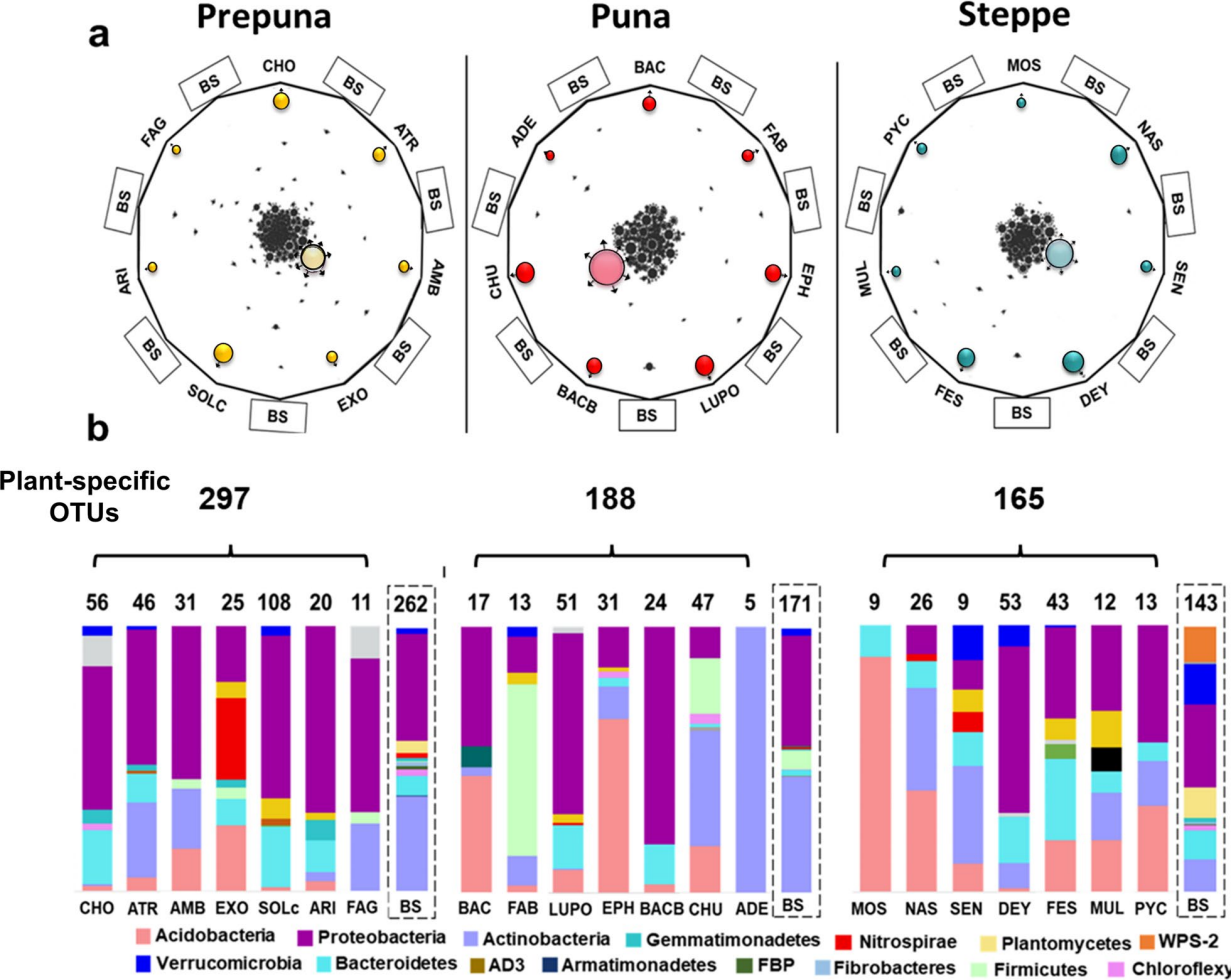
Common and plant-specific OTUs at the BS and the RSS compartments and their taxonomic composition. **a** In the Sungear polygons, the RSS and the BS compartments are organized in vertices of a polygon, which contains circles of different sizes that represent intersections and relative abundances of OTUs from each compartment. Dark colored circles represent plant-specific OTUs. Light color circles, in the center of the Sungear chart, represent OTUs from the 21 samples of the BS compartments. Black circles represent OTUs common to BS and RSS compartments. **b** The bars under each Sungear polygon show the taxonomic composition and the number of plant-specific OTUs of each plant in the three different vegetation belts (dark colors in **a**). The bar inside the dashed box shows the taxonomic composition of the nonredundant core of taxa identified in the 21 samples of BS compartments (light colors in **a**). The full names of the plant acronyms can be found in Additional file 7: Table S1

Comparison among soil bacterial communities by Venn diagram analysis showed that 37 to 49% of the OTUs were shared between the BS and the RSS at each vegetation belt (Fig. 2b). To determine the set of OTUs that were plant-specific (i.e., OTUs excluded from all other RSS and from the total BS samples at each vegetation belt; hereinafter plant-specific OTUs), a visualization of multiple comparisons of the OTU data was obtained using Sungear software [55] (Fig. 3a).

For example, of the 1003 prepuna OTUs, 741 were detected in the RSS compartment in at least one of the seven plants analyzed. When we compared the RSS OTUs among plants, 40% of these (297 OTUs) were plant specific. This percentage was reduced to 27% in puna and 24% in steppe (Additional file 7: Table S4). On average, the percentage of plant-specific OTUs per plant ranged from 5.7% (prepuna) to 3.5% (steppe). The relative abundances of prepuna OTUs ranged from 3.3% for *Fagonia chilensis* to 47.7% for *Solanum chilense* (Additional file 7: Table S4). In the puna, we found 188 plant-specific OTUs and *Adesmia erinacea* and *Lupinus oreophilus* had the lowest (1.6%) and highest (31.5%) relative abundances, respectively. Finally, in RSS from the steppe, we found 165 plant-specific OTUs; here, two members of the family Poaceae, *Calamagrostis crispa* and *Calamagrostis cabrerae,* had relative abundances of 26% and 16%, respectively (Additional file 7: Table S4).

When the taxonomic composition of plant-specific OTUs for each plant was examined, we found differences in taxa composition among plants of each vegetation belt, even at the phylum level (Fig. 3b). In general, members of Proteobacteria, Acidobacteria, Actinobacteria, and Gemmatimonadetes were the most abundant phyla within the plant-specific OTU communities. In particular, Proteobacteria species were dominant, accounting for more than 50% of the total plant-specific OTUs across all the RSS compartments. However, members of Nitrospirae, Firmicutes, and Plantomycetes were also found in some plants (Fig. 3b). We also identified a collection of OTUs that were present only in the BS compartment of each vegetation belt (Fig. 3a, light-colored circle in the center of the Sungear chart), which showed differences in their taxonomic composition (Fig. 3b, dashed box). Taken together, these results provide evidence of a plant-specific community composition in the RSS microbial communities and further support the notion that RSS introduces restrictions or promotes the recruitment of a subset of bacteria that colonize the rhizosphere from its surrounding BS.

### Prediction of bacterial taxa and chemical elements that distinguish between the BS and the RSS compartments using the random forest algorithm

**Fig. 4:**
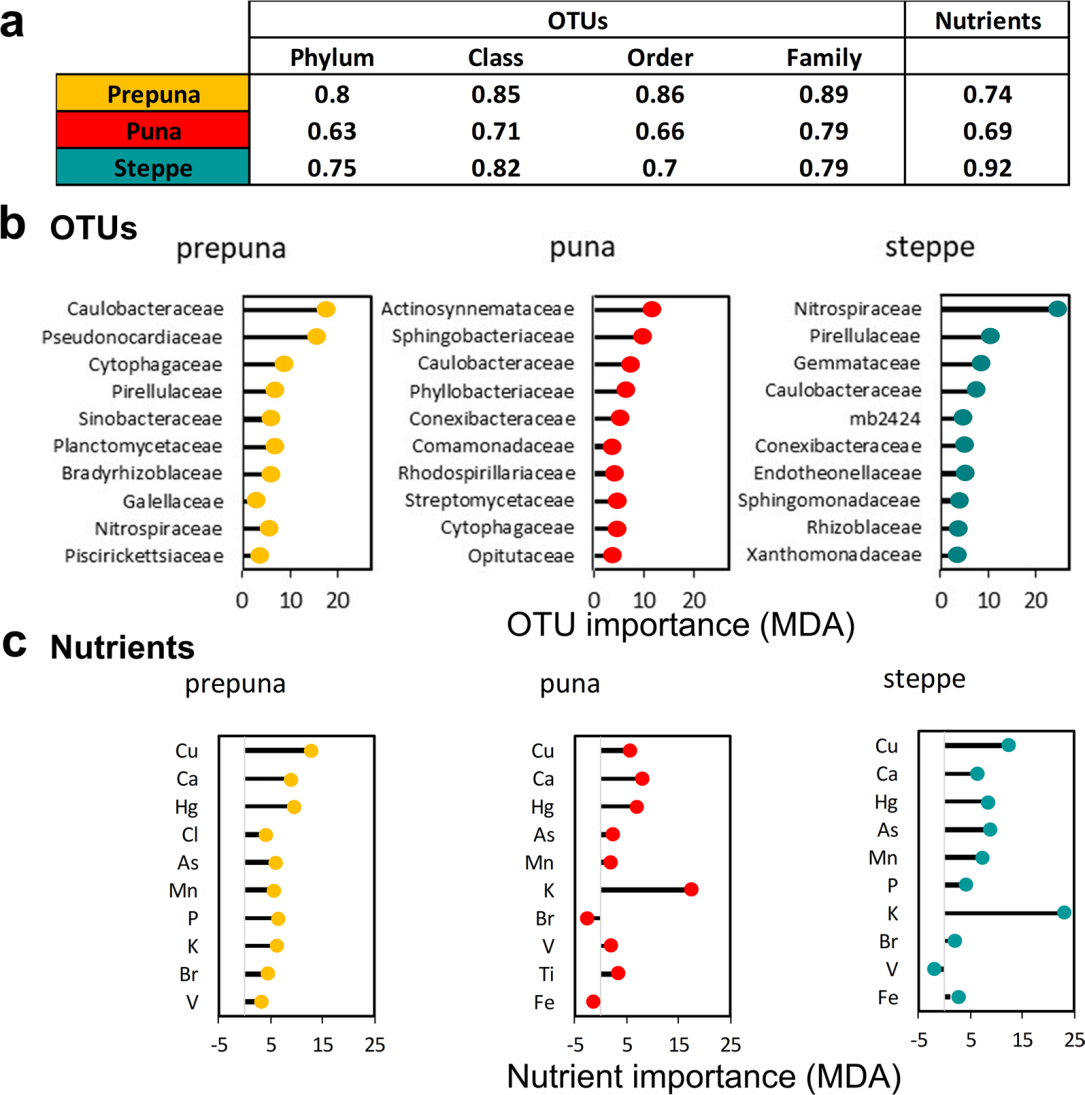
Best discriminant factors to differentiate between the BS and the RSS samples using machine learning. **a** 10CV accuracy of random forest classification models to discriminate between BS and RSS samples based on the relative abundance of OTUs or chemical component concentrations in different soil compartments at different taxonomic levels. **b** OTUs ranked from top to bottom, according to their importance in the random forest model (in terms of mean decrease in accuracy (MDA))- **c** chemical components

The next step was to apply the random forest algorithm to identify members of soil bacterial communities that could best represent the changes in the soil bacteria between the BS and the RSS across the altitudinal gradient. For each vegetation belt, the relative abundance of the OTUs was used to build models using phylum, class, order, or family levels, with the best accuracy achieved using the family level in all cases (Fig. 4a).

Interestingly, the most important predictor variables were different, although Caulobacteraceae appeared in the top four taxa of the vegetation belts (Fig. 4b). In the prepuna, the most important predictors were taxa of the families Caulobacteraceae and Pseudonocardiaceae belonging to the phyla Proteobacteria and Actinobacteria, respectively; in the puna, they were Actinosynnemataceae (Actinobacteria) and Sphingobacteriaceae (Bacteroidetes); and in the steppe, the were mainly Nitrospiraceae (Nitrospirae) (Fig. 4b). Overall, the ten most discriminating bacterial families between the BS and RSS compartments were not recurrent in the three vegetation belts, suggesting that the changes observed along the TLT depended more on the specific plant community composition than on only the transition from the BS to the RSS.

We repeated the random forest analysis to build a model that differentiated the BS from the RSS based on the chemical composition of the soils. We observed that except for the steppe, the accuracy of the predictive models was higher using the OTUs than the concentration of the chemical components (Fig. 4c). Nevertheless, to discriminate between the RSS and BS samples from the prepuna, the most important chemical variable for the random forest model was Cu, which was more concentrated in the RSS than in the BS samples (average concentration BS = 18 73 µg/L, RSS = 73 µg/; Additional file 7: Table S2). The most important variable to discriminate between compartments in the puna and steppe was K (Fig. 4c), which was also higher in the RSS in both vegetation belts (puna BS = 7.8 mg/L, RSS = 18.5 mg/L; steppe BS = 6.0 mg/L, RSS = 21.7 mg/L; Additional file 7: Table S2). The top ten most discriminating elements between the BS and RSS compartments exhibited differences in hierarchy and magnitude between the vegetation strata (Fig. 4c), a result that is in agreement with previous reports indicating that, despite the small spatial scale of the TLT, most nutritional variables changed along this altitudinal gradient [25, 45].

### Network analysis of the RSS and BS compartments at each vegetation belt

**Fig. 5:**
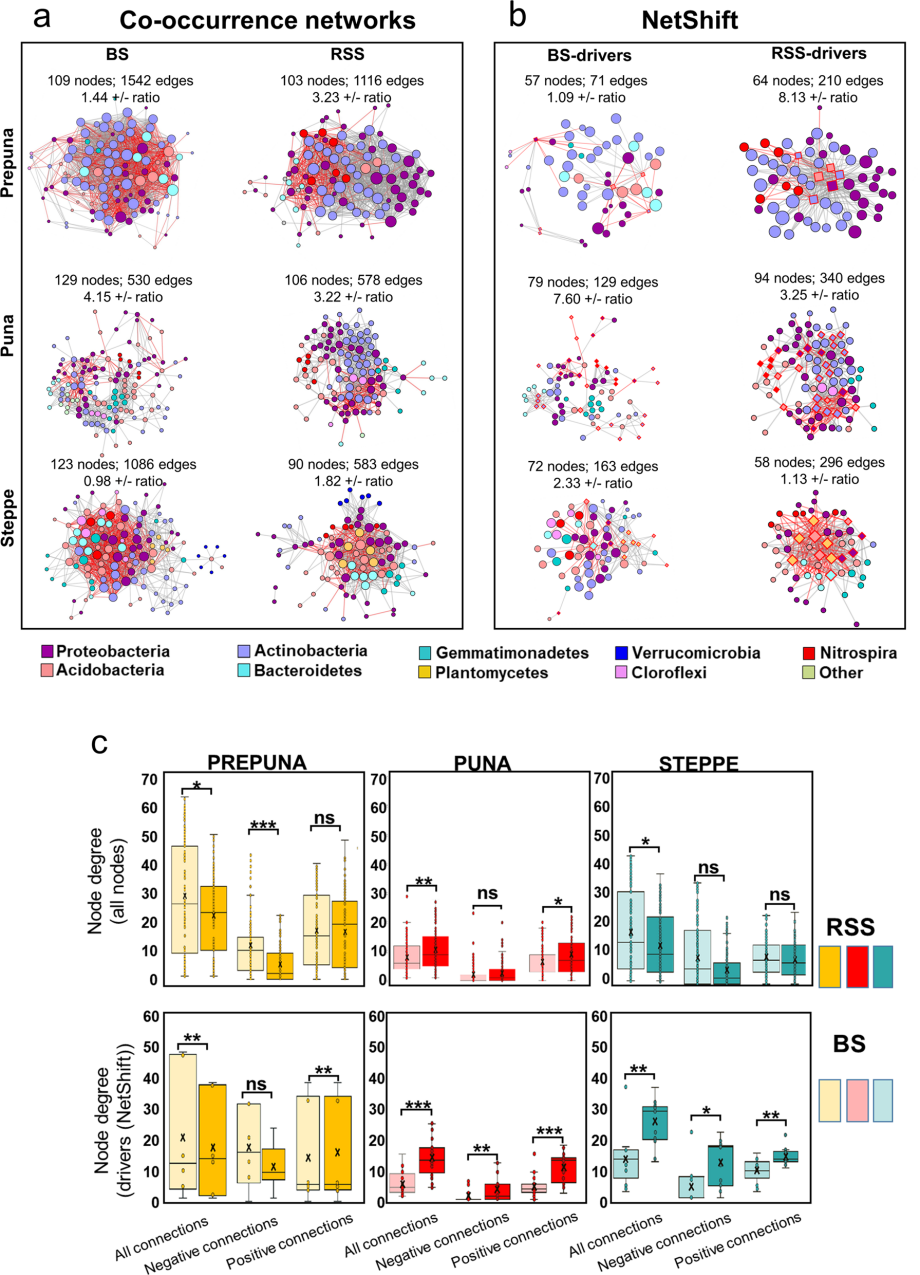
Complete and driver microbial co-occurrence networks of the BS and the RSS from the three vegetation belts. **a** Interactions were inferred from microbial OTU abundances. Each node represents an OTU or grouped taxa, and each edge represents a significant pairwise association between them (gray lines: copresences; red lines: exclusions). The different colors of nodes represent distinct phyla. Node sizes are proportional to the number of connections (degree) of each network (maximum node degree was 63). **b** In BS- and RSS-driver networks, drivers correspond to the red border diamond nodes. Statistical results of co-occurrence and NetShift networks are shown (number of nodes, edges and positive/negative ratios). **c** Box plots of the node’s degrees from all the connections, only from the positive connections and from the negative connections of the complete and drivers’ microbial co-occurrence BS and RSS networks. Statistically significant changes are noted as *p < 0.05; **p < 0.01; ***p < 0.001 using the Mann‒Whitney U test

The microbial interactions taking place in the BS and RSS bacterial communities of the different vegetation belts were examined by co-occurrence network analyses (Fig. 5a).

In this analysis, the nodes of the networks represent OTUs annotated at the phylum level, while the edges represent positive or negative correlations between the relative abundances of the OTUs. Based on the number of connections for each node (degree centrality), we observed that in each vegetation belt, the most connected OTUs, which are often proposed to be essential components of the bacterial community for network stability, were not the most abundant in either the BS or RSS compartment (Additional file 9: Table S5).

A global comparison of the RSS and BS networks indicated that the plant-associated microorganisms changed their interaction patterns compared to the surrounding BS microbiota, especially in the most extreme vegetation belts of the TLT. For instance, the RSS networks from the prepuna and steppe contained a lower number of nodes and edges than their BS counterparts, which, together with a lower clustering coefficient, indicated a less cohesive network (Fig. 5a, Additional file 7: Table S6). Considering all node connections, the node degree significantly declined from the BS to the RSS in the prepuna and steppe networks, while the puna showed an increase in the degree of the RSS network (Fig. 5c). These network parameters (number of nodes, node degree, number of edges, and clustering coefficient, Additional file 7: Table S6) suggest a decrease in the network complexity from the BS to the RSS in the most extreme vegetation belts of the TLT.

Regarding the network ratio of positive:negative connections, their values in the prepuna BS and RSS networks were 1.44 and 3.23, whereas the steppe BS and RSS networks displayed ratios of 0.98 and 1.82, respectively (Fig. 5a, Additional file 7: Table S6). In the prepuna, the increase in this ratio was associated with a significantly lower node degree of negative connections on RSS networks (Fig. 5c). In contrast, the puna had a higher positive:negative ratio in the BS than in the RSS network (4.15 and 3.22, respectively) (Fig. 5a, Additional file 7: Table S6) and a significantly higher node degree of positive connections in the RSS network (Fig. 5c). Hence, our network analysis indicated that in the most extreme belts from the TLT, the RSS compartments displayed a higher ratio of positive:negative links between members than plant-free soils. Additionally, we examined the number of positive and negative connections of nodes representing taxa with relative abundances greater than 1%. The data indicated that in the BS and RSS networks the prepuna and steppe, the number of nodes was higher and they had a greater number of positive and negative connections compared to the more abundant nodes from the BS and RSS networks of the puna (Additional file 7: Table S7). In addition, it was observed that these nodes exhibited a higher positive:negative connection ratio in the RSS network than in the BS network in all three vegetation belts (Additional file 7: Table S7).

To investigate the taxonomic and functional composition of the networks’ OTUs, we constructed abundance heatmaps and dendrograms at the phylum, class, and order levels and of the functions predicted by the KEGG pathways (Additional file 5: Figure S5). Overall, the taxonomic composition shows a similar pattern between the BS and the RSS in all belts (although with greater intensity in the puna), while the functional profiles between compartments differ considerably only in prepuna and steppe (Additional file 5: Figure S5, Additional file 10: Table S8). Given these observations, we performed a LEfSe analysis to identify differentially abundant functions between the BS and the RSS samples at each belt. This analysis revealed 71 KEGG pathways that differed significantly between the BS and RSS in the prepuna, 81 in the steppe and, as expected, none in puna (Additional file 7: Table S9). All these data suggest that plants in the most extreme belts of the TLT select more specific functions provided by the fraction of the bacterial community that they interact with.

### Potential driver taxa associated with the BS and the RSS networks

To identify potential taxa driving the transition from the BS to the RSS microbial communities, we used NetShift analysis [44], which allowed us to quantify the directional changes associated with each node (taxon) considering the BS as the control and the RSS as the case networks. Based on NetShift analysis, 6, 28 and 12 taxa were identified as potential drivers (NESH score > 1.8; DelBet > 0) within the prepuna, puna, and steppe networks, respectively (Additional file 6: Figure S6, Additional file 7: Table S10). To quantify the number of total, positive and negative connections of the driver taxa in their respective networks, we selected and examined the BS and RSS subnetworks containing only the nodes and edges associated directly with the set of driver taxa (Fig. 5b). As expected, in all cases, the total number of connections and average densities were higher in the RSS than in the BS driver networks, mainly due to an increase in the number of positive connections in the RSS networks (Additional file 7: Table S6). Regarding the ratio of positive:negative connections associated with driver taxa, the greatest change was observed in the prepuna, where the ratio increased from 1.1 to 8.1 in the transition from the BS to the RSS. On the other hand, in the puna and steppe, the ratio decreased from 7.6 to 3.3 and from 2.3 to 1.1, respectively (Additional file 7: Table S6). From the taxonomic point of view, the Actinobacteria taxa were exclusively from the prepuna network, along with two OTUs belonging to the class Chloracidobacteria and one member of the family Bradyrhizobiaceae (Additional file 7: Table S10). Actinobacteria1 and Actinomycetales were shared driver taxa between the prepuna and puna, while 11 taxa and 14 OTUs belonging to Rubrobacteraceae, Pseudonocardiaceae, Nitrospiraceae, Sphingomonadaceae, Bradyrhizobiaceae, and Hyphomicrobiaceae were specific to the puna network and classified as drivers (Additional file 7: Table S10). The steppe network shared one driver OTU from the class Chloracidobacteria with the puna network and displayed six taxa (Planctomycetes, Pirellulaceae, Planctomycetia, Pirellulales, Ellin6075, and Bosea) and six OTUs belonging to the families Ellin6075, Chitinophagaceae, Pirellulaceae, and Bradyrhizobiaceae as potential driver taxa (Additional file 7: Table S10).

## Discussion

Previous studies on the soil microbiota along this altitudinal gradient have shown that soil fertility is associated with both plant cover and microbial diversity [34, 36, 60]. In addition, plant soil-associated microbiota from the TLT have been characterized to understand the biotic factors that could influence their ability to survive in this harsh environment [25, 26]. Knowing that plant‒microbe interactions involve mechanisms by which microbes act beneficially in promoting plant growth, such as nitrogen fixation, protection against pathogens and drought resistance, among others [25, 26, 35], the above works suggest that a modulation of soil microbial diversity may be a characteristic of plants that are highly tolerant to diverse and extreme environments. Although we acknowledge here that each plant species could affect the microbial community in the RSS compartment [35], the main goal of the present work was to analyze plant effects at the plant community level.

### Differential impact of plant community on the soil microbial composition in different vegetation belts

Prepuna is the lowest vegetation belt, where only plants such as cushion cacti and small subshrubs such as *Atriplex imbricate* and *Fagonia chilensis* (Fig. 1, Additional file 7: Table S1), which can tolerate the dry conditions of this zone, are present [34, 43]. One of the mechanisms described in other species of *Atriplex* is the accumulation of high salt concentrations within their cellular vacuoles, which helps to mitigate water stress [61]. On the other hand, species of *Fagonia* have been shown to be great accumulators of carotenoids, total soluble sugars, sucrose, and proline to overcome drought stress [62]. Despite their own mechanisms to tolerate drought, these plants seem to recruit specific soil bacterial communities with specific functions that might help them deal with local environmental conditions [20, 29, 63]. The plant strategy of microbial recruitment might account for the major differences that we observed in the microbial communities associated with the BS and the RSS of the prepuna plant species. Accordingly, taxonomic analysis also showed that the prepuna is the vegetation belt with the highest number of plant-specific OTUs (n = 297 OTUs), suggesting that plant species in this vegetation belt have developed mechanisms to recruit specific microbial communities to cope with multiple adverse environmental conditions. For instance, there are different rhizobacterial genera that have been associated with phytoremediation, such as *Azospirillum* [64], from which we identified 96 OTUs in the RSS microbial community of the prepuna and none in that of the BS community (Additional file 8: Table S3). Similarly, the RSS samples from *Hoffmannseggia doellii* of the prepuna showed higher bacterial diversity indices than those of the puna samples, which suggests a link between *H. doellii*-associated bacterial soil diversity and the challenging environmental conditions faced by this plant [26].

In sharp contrast, the random forest model of the steppe, the highest vegetation belt, which is characterized by cushion plants, subshrubs, and perennial bunch grasses such as *Nassella nardoides* or *Calamagrostis crispa*, showed that the most important nutritional variable to discriminate between RSS and BS samples was K (Fig. 4). Even though K was also the best discriminant of the puna, its importance as a variable in the random forest model of the steppe was considerably higher. This observation might be related to the decreased soil pH over the TLT, which broadly affects nutrient solubility and availability [25, 34, 43, 60]. Moreover, it is well known that K is an essential nutrient that impacts several physiological and biochemical processes [65] that are also involved in plant resistance to biotic and abiotic stresses such as drought or extreme temperatures [66] and that several rhizospheric K-solubilizing microbes such as *Bacillus*, *Pseudomonas*, and *Aspergillus* expel organic acids, which solubilize insoluble K and make it available to plant roots.

Regarding the taxa predictors of the BS and the RSS compartments, *Caulobacteraceae* was in the top four taxa of all the vegetation belts (Fig. 4b), which is interesting considering that members from this family are copiotrophic [67] despite inhabiting this overall extreme environment. In the prepuna, the other most important predictor was the family Pseudonocardiaceae, which has often been observed in extreme environments, such as arid soils and stone surfaces [68]. Additionally, some of their representatives produce bioactive compounds with antimicrobial activity and thrive under strong UV-B irradiation [69]. Interestingly, in the steppe, the other most extreme environment from the TLT, Nitrospiraceae was the most frequently detected family (Fig. 4b), members of which are known key nitrite oxidizers [70]. Thus, these taxa could be important collaborators for the establishment of plant cover in desert soil, and their shift in abundance may be explained precisely by their particular features [71]. In general, our results indicate that both host species and their TLT distribution contributed to shaping the plant-specific microbial communities, as has been suggested in studies on sympatric alpine plant species [72].

### Differential impact of plant communities on the TLT co-occurrence microbial networks

The results from our network co-occurrence analysis showed differences between network complexity in the different soil compartments at the extremes of the TLT. For instance, the RSS networks from the prepuna and steppe showed a lower number of edges and degree of clustering between members than their BS counterparts. Furthermore, at these belts, the RSS networks displayed higher positive:negative ratios compared to the BS networks, a result that was not observed at the puna belt (Additional file 7: Table S6). The higher number of positive than negative connections is assumed to represent cooperative metabolism that could provide health benefits for the host since the competition between microbes can severely reduce the efficiency of any cooperative metabolism in favor of the host [73–76]. Thus, our data suggest that at the extreme environments of the gradient, plant communities are important factors that shift bacterium‒bacterium co-occurrence patterns toward less complex networks but with increased positive interactions among them [77]. This could be a strategy that favors soil bacterial communities growing in these hostile sites when associated with the plant root system, a characteristic also noted by [76] in the hyperarid Namib Desert. Consistently, this strategy appears less apparent in more favorable environments in the transect, such as the puna vegetation belt, where MAT and MAP conditions are less stressful in comparison to the prepuna and steppe belts, and where network analysis showed that positive connections dominated the BS and RSS bacterial communities.

In addition, considering the competitive exclusion principle [78], which proposes that the abundance of a population may reflect its ability to compete with members of other populations [79], we examined the number of positive and negative connections of nodes representing taxa with relative abundance greater than 1. The BS and RSS networks of the prepuna and steppe displayed a greater number of positive and negative connections than the more abundant nodes from the BS and RSS networks of the puna. Additionally, these nodes exhibited a higher positive:negative connection ratio in the RSS network than in the BS network in all three vegetation belts (Additional file 7: Table S7). Thus, these results indicate that the most abundant taxa of the co-occurrence networks preferentially established cooperative associations, a phenomenon more evident in the prepuna and steppe and in the soil compartment associated with plant roots. Therefore, the potential interactions of members of the microbial communities from the TLT did not support the stress gradient hypothesis, which proposes that the frequency of positive interactions between species increases with environmental stress [37] In addition, each plant community appears to moderate the stress gradient, increasing the efficiency of the soil microbial community mainly at the highest and lowest elevations of the TLT, where environmental conditions are less suitable for the plant community (lowest temperature and highest aridity, respectively). Consistent with these findings, the functional analysis of the OTUs of the networks showed that none of the predicted functions present in the puna differed between the BS and the RSS but did differ among the prepuna and steppe compartments, suggesting that plants select specific functional capacities in the more extreme environments of the transect.

### Identification of specific driver taxa during the transition from the BS to the RSS microbial communities

The changes between co-occurrence networks corresponding to the BS and the RSS in each vegetation belt were examined using the NetShift method [44], which allowed us to explore differences among taxa connections of each compartment pair (BS and RSS), as well as to compare their global and local graph properties, a process that is independent of any external biological observation [80]. Although no driver OTUs or taxa were shared between the three vegetation belts, OTUs belonging to the order Rhizobiales, including members of the families Bradyrhizobiaceae and Hyphomicrobiaceae, displayed a high NESH score as potential driver taxa in the prepuna, puna, and steppe (Additional file 7: Table S10). Genera of these families have been shown to contain soil microorganisms that can establish beneficial relationships with plant roots by fixing nitrogen [81, 82], including OTUs belonging to *Bosea* [83], *Bradyrhizobium* [84, 85] and *Rhodoplanes* [86]. Taxa and specific OTUs known to include species relevant for plant protection were also identified as potential drivers of the transition between the BS to the RSS compartment, particularly in the puna and steppe. Some of these taxa were Sphingomonadaceae [84, 87] with OTUs belonging to the genus *Kaistobacter* involved in the degradation of aromatic compounds [88], together with plant growth promoter taxa such as Nitrospiraceae [89, 90], Rubrobacteraceae [91] and Pseudonocardiaceae [71] in the puna and Pirellulaceae [92, 93] and Chitinophagaceae [94, 95] in the steppe. Thus, these driver taxa appear to be good candidates as indicators not only of environmental perturbations but also of candidate bacteria with plant beneficial traits along the TLT.

We found a difference between the BS and the RSS nutrient correlations (Additional file 2: Figure S2) that could indicate that members of the bacterial community are selected by plants in the RSS compartment to modify nutrient levels and their availability in the soil. This suggests that local variation in abiotic environmental conditions within the RSS compartment of each TLT vegetation belt was a possible driver of differential composition and pattern of co-occurrence relations within the soil microbial community associated with the root system compared with the bulk soil. In addition, our results indicated that a portion of the bacterial community was exclusively associated with each plant species within the vegetation belts (Fig. 3; Additional file 3: Figure S3). Most likely, modifications in the abundance of soil bacteria in the RSS and changes in their ecological interactions with respect to the BS might have also been affected by root secretions, plant litter, or plant secondary metabolites in a genotype-specific manner, which have been reported as key factors in controlling the assembly of root-associated microbial communities [94, 96–98].

## Conclusions

We identified a complex plant-microbial community along an altitudinal gradient in the Atacama Desert. Overall, our results did not support the stress gradient hypothesis because the number of positive interactions did not increase under higher stress environments of the TLT gradient. However, in the RSS compartment, where the plant community appears to moderate the abiotic stress gradient, the efficiency of the soil microbial community increased compared to the BS, suggesting that positive or negative interactions may be context dependent. These results contribute to our understanding of how the natural plant community assembles and the abiotic environment interplays with the structure of plant-associated microbial communities, an important biodiversity facet that is often ignored but that can play key roles in the response of plant communities to ongoing climate change.

## Supplementary Information

**Additional file 1: Figure S1.** Location of the study site and sampling procedure. **a** The site at which the study and sampling were performed was located in northern Chile (left panel) in the Andes Mountains near Atacama Salar (right panel). The colored dots indicate the geographic positions of the prepuna (orange dot), puna (red dot) and steppe (green dot). **b** Two different soil compartments were sampled: RSS (root surrounding soil: bacteria loosely attached to the roots) and BS (bulk soil). **c** At each vegetation belt: (i) RSS was sampled in triplicate (three biological replicates of each plant species (Sp1-R1; Sp1-R2; Sp1-R3)); (ii) BS samples were collected in triplicate (BS-R1, BS-R2, BS-R3) at 10 cm depth from the ground and at least 1 m away from each sampled plant. This image is an example of the sampling procedure that was performed for the 21 plant species included in the study.

**Additional file 2: Figure S2.** Taxonomic composition of the microbiomes along the Talabre-Lejía transect. Taxonomic composition of the microbiomes in the three vegetation belts. Each bar represents one of the triplicates of the phyla relative abundances in the BS and the RSS compartments. Plant family classification is indicated for each plant species.

**Additional file 3: Figure S3.** Correlation between soil components in the RSS and BS. Correlogram showing the Pearson's correlation patterns between mean concentrations of selected nutrients and chemical components measured in both compartments in the three analyzed vegetation belts. Blue circles represent a positive correlation, while red circles represent a negative correlation. The size of the circles indicates the strength of the correlation.

**Additional file 4: Figure S4.** Richness and diversity analysis between the BS and the RSS of the soil microbiota. Boxplots show the richness and diversity indices (from left to right: Shannon and phylogenetic diversity (PD)) between BS and RSS samples from **a** the entire TLT and **b** each vegetation belt.

**Additional file 5: Figure S5.** Taxonomic composition and predicted functions of the networks’ OTUs. In the upper panel, a heatmap shows the relative abundance of the taxonomic composition of the networks’ OTUs at the phylum, order, and class levels. In the lower panel, a heatmap shows the relative abundance of the functional prediction of KEGG pathways performed with Picrust2. From left to right, a comparison between BS and RSS from the prepuna, puna, and steppe.

**Additional file 6: Figure S6.** Network view of the community shuffle plot detected using the neighbor shift (NetShift). Changes between core OTUs from the BS and the RSS co-occurrence networks from prepuna, puna, and steppe were detected using NetShift methodology. Nodes of the core taxa are arranged on the periphery of the circle. Node sizes are proportional to their scaled NESH score (Additional file 10: Table S8). Red nodes represent increases in their betweenness from the BS to the RSS. Therefore, red nodes represent potential “driver taxa”. The connections of the nodes in green are edges present only in the BS network, those in red are only present in the RSS, and those in blue are present in both compartments. The different colors of node labels were randomly assigned.

**Additional file 7: Table S1.** Distribution across altitudinal gradient and characteristics of TLT plants. **Table S2.** Environmental and physicochemical variables. **Table S4.** Number of OTUs belonging to RSS, RSS-Non-core, BS and BS Non-core for each plant for each plant in the three vegetation belts. **Table S6.** Microbial co-occurrence networks' parameters. **Table S7.** Edge properties of abundant nodes in the Networks. **Table S9.** LEfSe analysis of differences between BS and RSS. **Table S10.** Summary of the node properties calculated by NetShift.

**Additional file 8. Table S3.** OTUs frequency in each sample of prepuna, puna and steppe.

**Additional file 9. Table S5.** Network's nodes.

**Additional file 10. Table S8.** KEGG pathways predicted with Picrust2.

## Data Availability

All data is directly attached to this paper. In addition, metabarcoding data can be found at NCBI public repository under the BioProject accession number PRJNA489888. Universidad de Concepción's Herbarium (CONC) houses a voucher of each of the plant species surveyed in the Talabre-Lejia transect.
