# Supplementary figures and images for: Testing the stress gradient hypothesis in soil bacterial communities associated with vegetation belts in the Andean Atacama Desert
Source: Environ Microbiome. 2023 Mar 28;18:24. doi: 10.1186/s40793-023-00486-w (PMC10052861; doi:10.1186/s40793-023-00486-w)

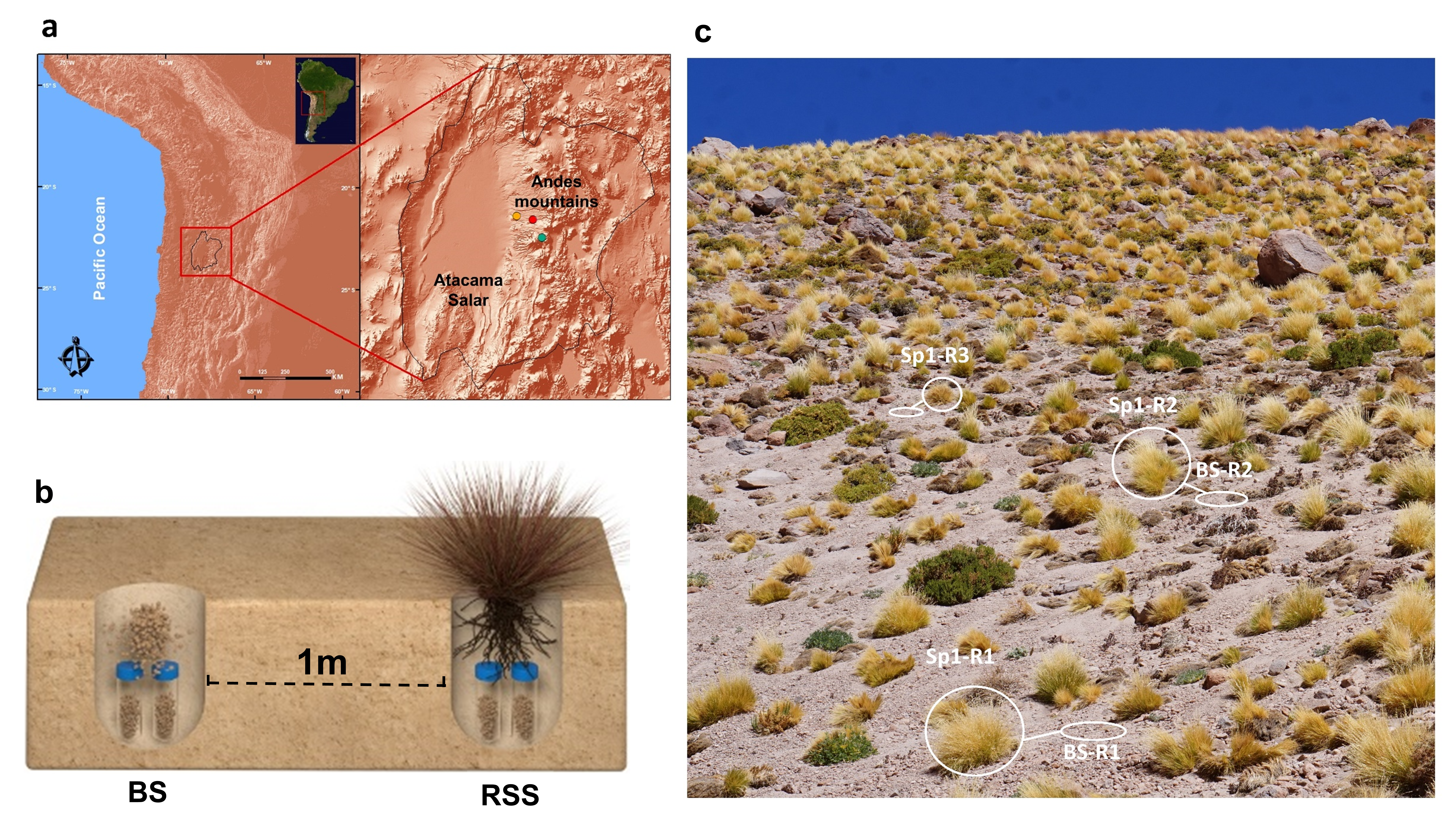

Supplement: Supplementary file 1 — Additional file 1: Figure S1. Location of the study site and sampling procedure. a The site at which the study and sampling were performed was located in northern Chile (left panel) in the Andes Mountains near Atacama Salar (right panel). The colored dots indicate the geographic positions of the prepuna (orange dot), puna (red dot) and steppe (green dot). b Two different soil compartments were sampled: RSS (root surrounding soil: bacteria loosely attached to the roots) and BS (bulk soil). c At each vegetation belt: (i) RSS was sampled in triplicate (three biological replicates of each plant species (Sp1-R1; Sp1-R2; Sp1-R3)); (ii) BS samples were collected in triplicate (BS-R1, BS-R2, BS-R3) at 10 cm depth from the ground and at least 1 m away from each sampled plant. This image is an example of the sampling procedure that was performed for the 21 plant species included in the study. [file 40793_2023_486_MOESM1_ESM.tif]

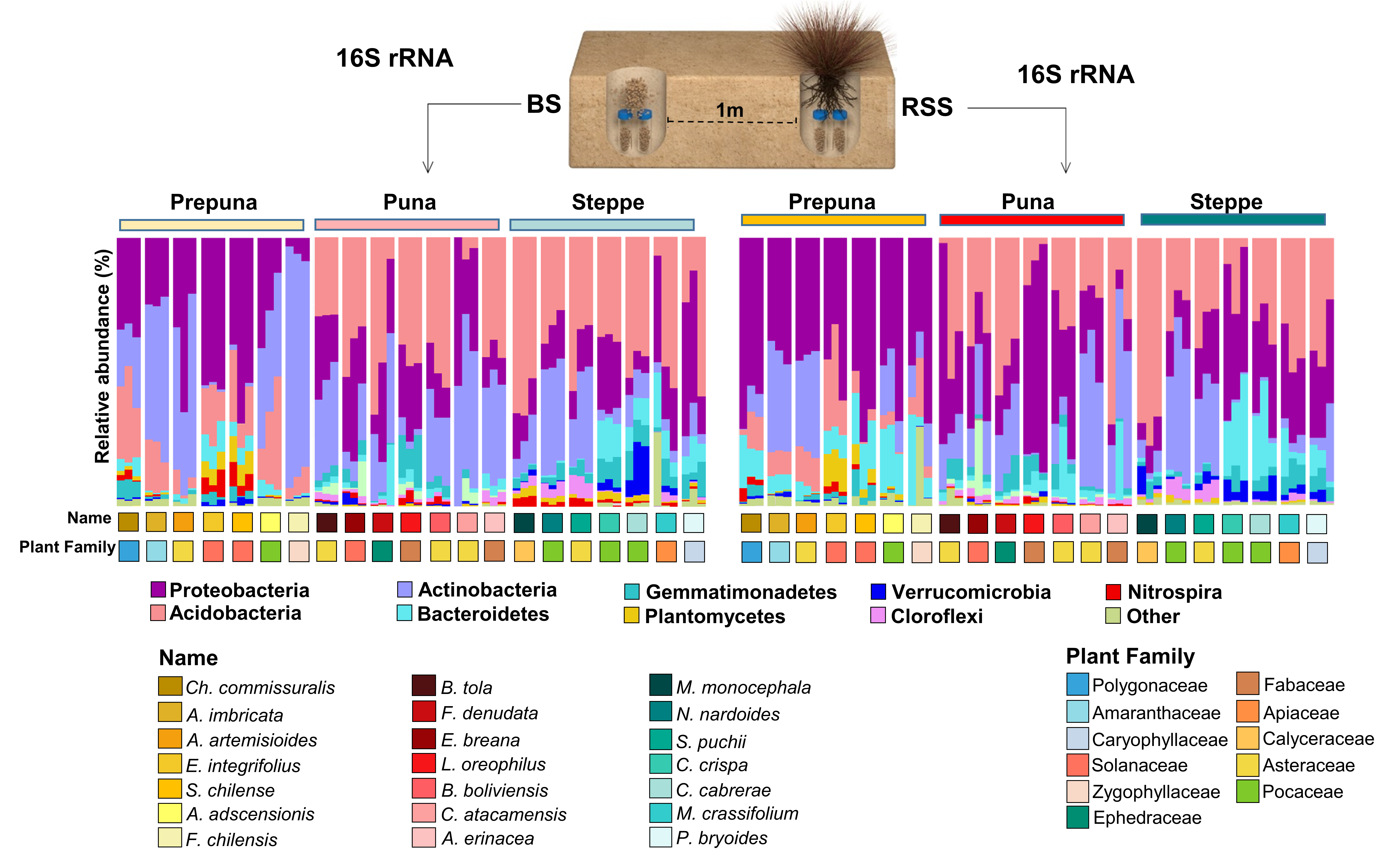

Supplement: Supplementary file 2 — Additional file 2: Figure S2. Taxonomic composition of the microbiomes along the Talabre-Lejía transect. Taxonomic composition of the microbiomes in the three vegetation belts. Each bar represents one of the triplicates of the phyla relative abundances in the BS and the RSS compartments. Plant family classification is indicated for each plant species. [file 40793_2023_486_MOESM2_ESM.tif]

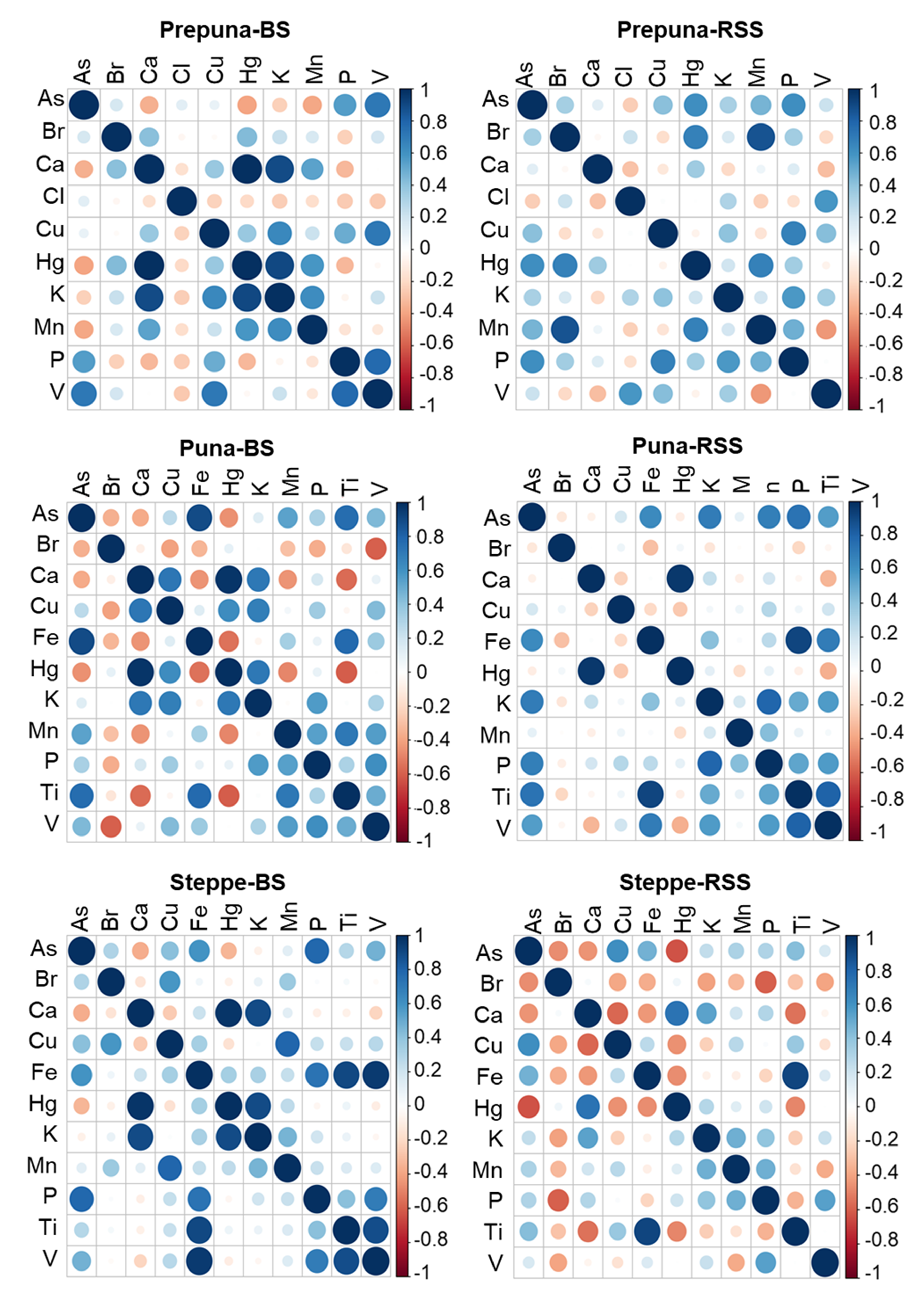

Supplement: Supplementary file 3 — Additional file 3: Figure S3. Correlation between soil components in the RSS and BS. Correlogram showing the Pearson's correlation patterns between mean concentrations of selected nutrients and chemical components measured in both compartments in the three analyzed vegetation belts. Blue circles represent a positive correlation, while red circles represent a negative correlation. The size of the circles indicates the strength of the correlation. [file 40793_2023_486_MOESM3_ESM.tif]

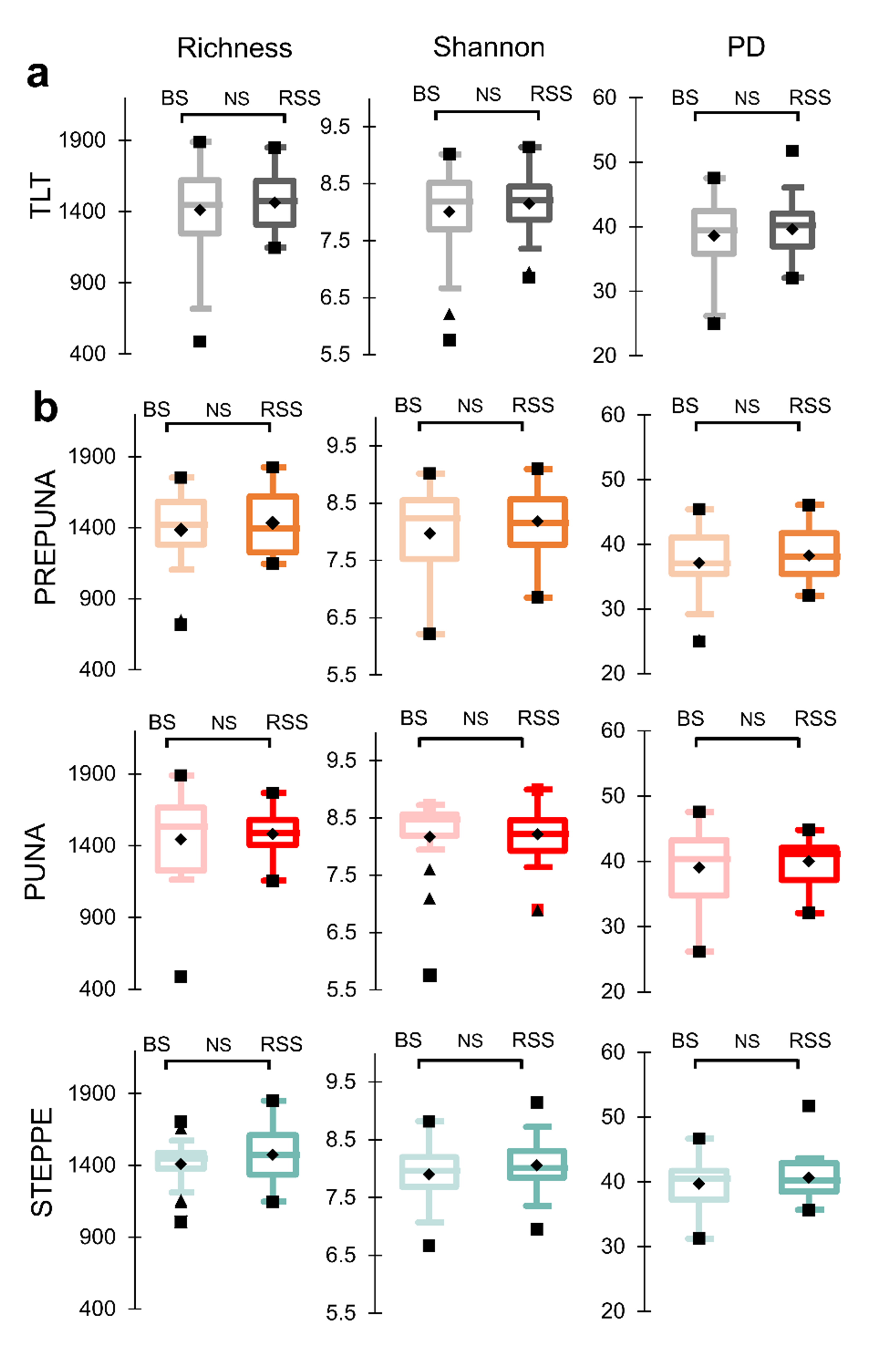

Supplement: Supplementary file 4 — Additional file 4: Figure S4. Richness and diversity analysis between the BS and the RSS of the soil microbiota. Boxplots show the richness and diversity indices (from left to right: Shannon and phylogenetic diversity (PD)) between BS and RSS samples from a the entire TLT and b each vegetation belt. [file 40793_2023_486_MOESM4_ESM.tif]

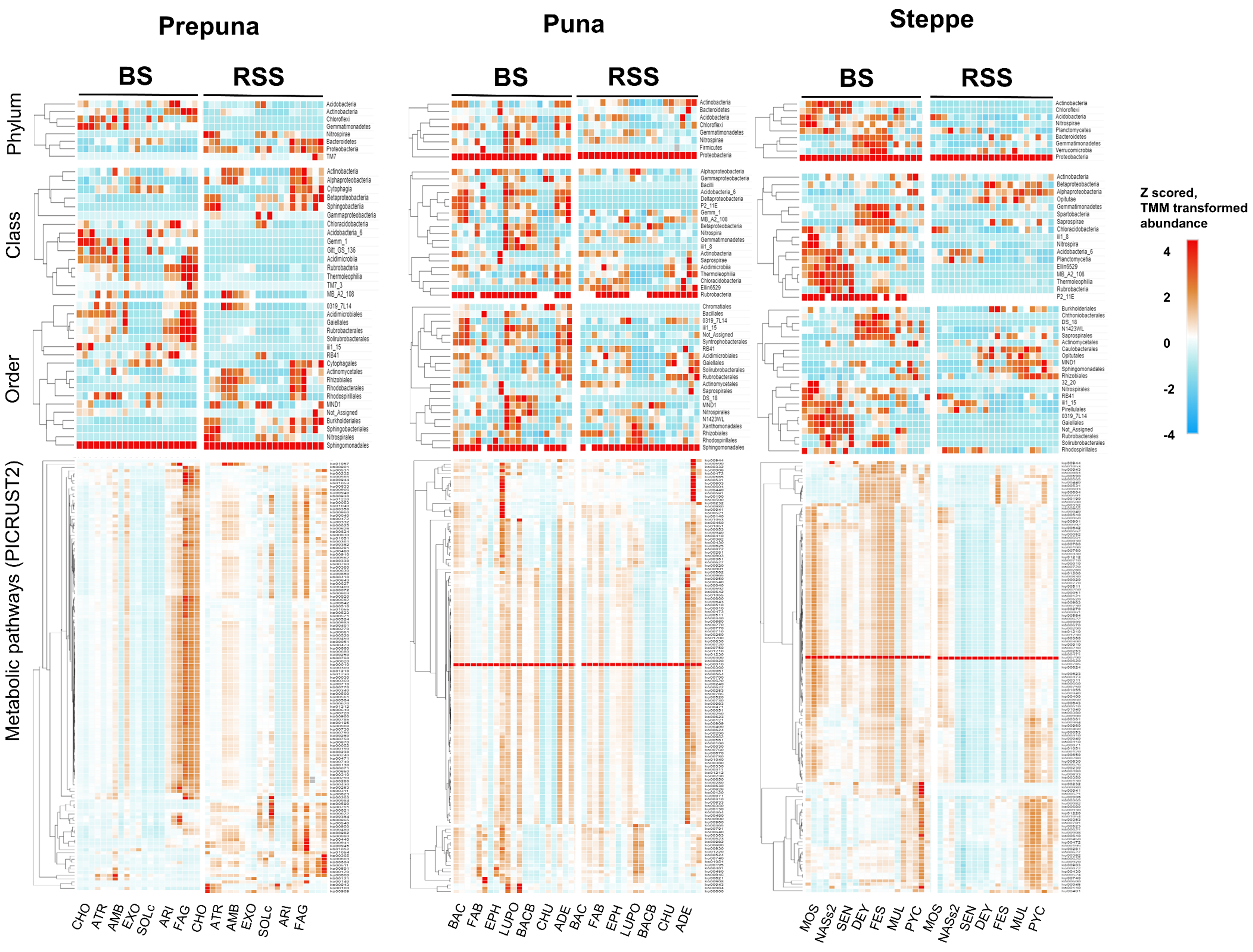

Supplement: Supplementary file 5 — Additional file 5: Figure S5. Taxonomic composition and predicted functions of the networks’ OTUs. In the upper panel, a heatmap shows the relative abundance of the taxonomic composition of the networks’ OTUs at the phylum, order, and class levels. In the lower panel, a heatmap shows the relative abundance of the functional prediction of KEGG pathways performed with Picrust2. From left to right, a comparison between BS and RSS from the prepuna, puna, and steppe. [file 40793_2023_486_MOESM5_ESM.tif]

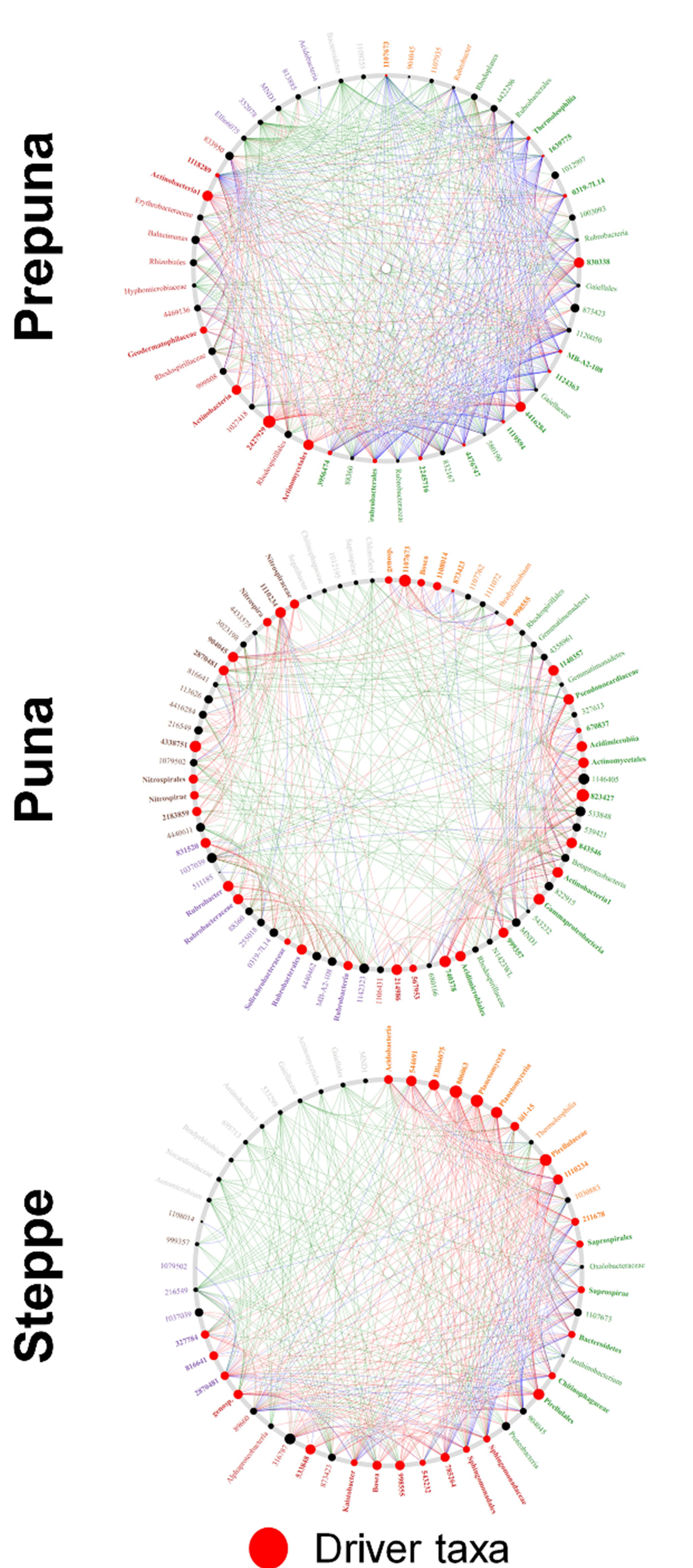

Supplement: Supplementary file 6 — Additional file 6: Figure S6. Network view of the community shuffle plot detected using the neighbor shift (NetShift). Changes between core OTUs from the BS and the RSS co-occurrence networks from prepuna, puna, and steppe were detected using NetShift methodology. Nodes of the core taxa are arranged on the periphery of the circle. Node sizes are proportional to their scaled NESH score (Additional file 10: Table S8). Red nodes represent increases in their betweenness from the BS to the RSS. Therefore, red nodes represent potential “driver taxa”. The connections of the nodes in green are edges present only in the BS network, those in red are only present in the RSS, and those in blue are present in both compartments. The different colors of node labels were randomly assigned. [file 40793_2023_486_MOESM6_ESM.tif]
